# Evaluation of Downstream Processing, Extraction, and Quantification Strategies for Single Cell Oil Produced by the Oleaginous Yeasts *Saitozyma podzolica* DSM 27192 and *Apiotrichum porosum* DSM 27194

**DOI:** 10.3389/fbioe.2020.00355

**Published:** 2020-04-24

**Authors:** Olga Gorte, Rebecca Hollenbach, Ioannis Papachristou, Christian Steinweg, Aude Silve, Wolfgang Frey, Christoph Syldatk, Katrin Ochsenreither

**Affiliations:** ^1^Institute of Process Engineering in Life Science 2: Technical Biology, Karlsruhe Institute of Technology, Karlsruhe, Germany; ^2^Institute for Pulsed Power and Microwave Technology, Karlsruhe Institute of Technology, Karlsruhe, Germany; ^3^Institute of Process Engineering in Life Science 3: Bioprocess Engineering, Karlsruhe Institute of Technology, Karlsruhe, Germany

**Keywords:** single cell oil, oleaginous yeasts, downstream processing, cell disruption, lipid extraction, transesterification, *Saitozyma podzolica* DSM 27192, *Apiotrichum porosum* DSM 27194

## Abstract

Single cell oil (SCO) produced by oleaginous yeasts is considered as a sustainable source for biodiesel and oleochemicals since its production does not compete with food or feed and high yields can be obtained from a wide variety of carbon sources, e.g., acetate or lignocellulose. Downstream processing is still costly preventing the broader application of SCO. Direct transesterification of freeze-dried biomass is widely used for analytical purposes and for biodiesel production but it is energy intensive and, therefore, expensive. Additionally, only fatty acid esters are produced limiting the subsequent applications. The harsh conditions applied during direct esterification might also damage high-value polyunsaturated fatty acids. Unfortunately, universal downstream strategies effective for all yeast species do not exist and methods have to be developed for each yeast species due to differences in cell wall composition. Therefore, the aim of this study was to evaluate three industrially relevant cell disruption methods combined with three extraction systems for the SCO extraction of two novel, unconventional oleaginous yeasts, *Saitozyma podzolica* DSM 27192 and *Apiotrichum porosum* DSM 27194, based on cell disruption efficiency, lipid yield, and oil quality. Bead milling (BM) and high pressure homogenization (HPH) were effective cell disruption methods in contrast to sonification. By combining HPH (95% cell disruption efficiency) with ethanol-hexane-extraction 46.9 ± 4.4% lipid/CDW of *S. podzolica* were obtained which was 2.7 times higher than with the least suitable combination (ultrasound + Folch). *A. porosum* was less affected by cell disruption attempts. Here, the highest disruption efficiency was 74% after BM and the most efficient lipid recovery method was direct acidic transesterification (27.2 ± 0.5% fatty acid methyl esters/CDW) after freeze drying. The study clearly indicates cell disruption is the decisive step for SCO extraction. At disruption efficiencies of >90%, lipids can be extracted at high yields, whereas at lower cell disruption efficiencies, considerable amounts of lipids will not be accessible for extraction regardless of the solvents used. Furthermore, it was shown that hexane-ethanol which is commonly used for extraction of algal lipids is also highly efficient for yeasts.

## Introduction

Microbial triacylglycerols (SCO) which are produced by oleaginous bacteria, algae, yeast, and fungi are promising sustainable platform chemicals. SCOs are chemically equivalent to plant oils, but can be produced independent of season, climate and location using a wide range of cheap and abundant carbon sources including waste streams from food and other agricultural based industries (Yousuf et al., 2010; Ling et al., 2013; Kot et al., 2017) or renewable carbon sources (Alvarez et al., 1992; Liu et al., 2015; Fei et al., 2016) and therefore do not compete with food or feed. In particular, SCO production with yeasts is advantageous because of their fast growth rate and greater convenience to scale up cultivation than that of autotrophic microalgae since no light is needed (Li et al., 2008; Ageitos et al., 2011; Ochsenreither et al., 2016). Oleaginous yeast lipids can be used to produce biodiesel, and might find application in food industry and as building blocks for biopolymers (Vasconcelos et al., 2019). Pilot plant scale production of yeast SCO increases the potential of these microorganisms. Xue et al. (2010) cultivated *Rhodotorula glutinis* on starch wastewater in a 300 L scale and reached 35% lipid content. Moreover, Soccol et al. (2017) successfully processed a fed-batch strategy using sugarcane juice with *Rhodosporidium toruloides* in a 1000 L plant. Thereby, a 6.3 fold higher biodiesel yield was reached compared to standard biodiesel from soybean.

Despite all these advantages, commercial SCO production is restricted to high-value oils containing high amounts of polyunsaturated fatty acids for nutritional purposes (Ratledge, 2004; Mendes et al., 2009; Ji et al., 2014). Production of SCO resembling plant oils is currently too expensive to allow commercialization (Ratledge and Cohen, 2008; Leong et al., 2018). One major obstacle is still the efficient extraction and purification of SCO since high costs arise from energy and labor expenditure to recover the intracellular lipids. The rigidity and robustness of the yeast cell wall mainly contributes to the resistance to organic solvents. These circumstances have an evolutionary background to protect and adapt the cell to environmental conditions by building backbones of cross-linked glucan fibers, mannoprotein, and chitin (Phaff, 1971; Jacob, 1992). On this account, an efficient cell disruption is mandatory for successful lipid recovery and needs to be optimized for each yeast strain, since the cell wall composition may vary considerably between species. In literature lots of attempts using various methods and different species can be found (Jin et al., 2012; Zhang et al., 2014; Meullemiestre et al., 2016; Vasconcelos et al., 2018). However, rating the methods is difficult, as reported results of different laboratories may not be comparable. Although the importance of cell disruption for lipid extraction efficiency has been recognized (Rakesh et al., 2015; Ramola et al., 2019), cell disruption is usually not determined and therefore, thresholds for effective extractions have not been communicated.

**FIGURE 1:**
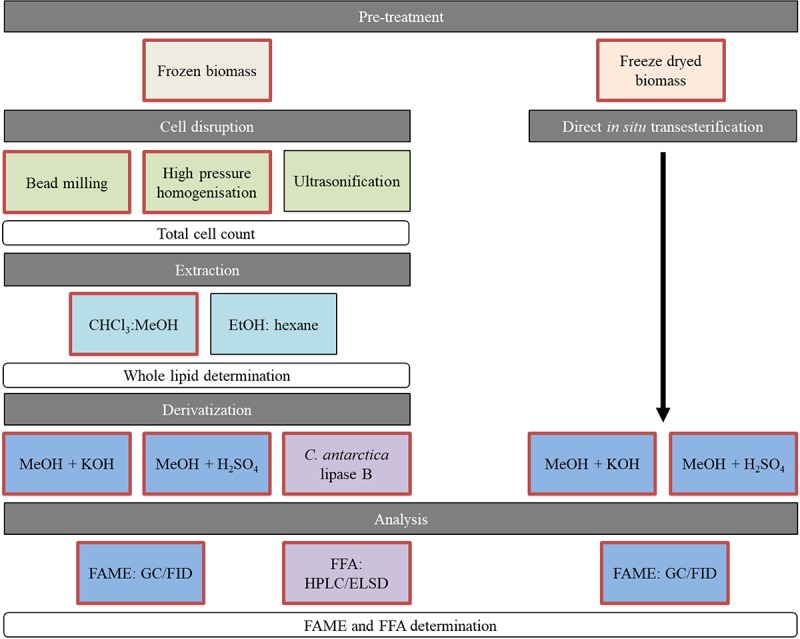
Overview of performed methods for lipid recovery. Red framed boxes were analyzed for both yeasts. The black framed methods were implemented on *S. podzolica* only. FAMEs, fatty acid methyl esters; FFAs, free fatty acids.

In this study, we evaluate combinations of cell disruption and extraction methods on two by Schulze et al. (2014) isolated oleaginous yeasts, *Saitozyma podzolica* DSM 27192 and *Apiotrichum porosum* DSM 27194. These unconventional yeasts have been recently characterized as co-producer of intracellular SCO and extracellular gluconic acid, when cultivated on glucose (Schulze et al., 2014; Qian et al., 2019). Additionally, Qian et al. (2020) demonstrated that both yeasts are robust SCO producers capable of using the cheap industrial by-product acetate. However, the interest in these yeasts was only focused on upstream processing. We are the first to provide any kind of insights into lipid downstream methods processed with these new yeasts including an extensive evaluation. The workflow is illustrated in Figure 1. Mechanical cell disruption methods, namely bead-milling (BM) (mechanical disruption by grinding), HPH (mechanical disruption by pressure) and ultrasonification (U) (mechano-physical disruption by cavitation) were implemented because of their great industrial potential and the use at a large scale in other industries (Chisti and Moo-Young, 1986; Rodríguez-Alcalá et al., 2009; Ochsenreither et al., 2016). These disruption methods were applied on frozen, wet yeast biomass prior to chloroform methanol extraction according to Folch (F) (Folch et al., 1957) and Bligh and Dyer (BD) (Bligh and Dyer, 1959), which are the most popular lipid extraction methods in literature and known for their efficiency (Schmid et al., 1973) in lipid recovery at laboratory-scale. Additionally, the ethanol and hexane (EH) system, which is commonly used in food industry (Grima et al., 1994; Cheng and Rosentrater, 2017), was tested in terms of whole lipid extraction yield. The extracted whole lipids, including storage and membrane lipids are applicable for oleochemical building blocks production.

Further investigations were performed on lipid profiles after enzymatic hydrolysis to FFAs and transesterification to FAME via HPLC-ELSD and GC-FID, respectively. All results were also compared with the lipid yield and profile from direct transesterification (DT) of freeze-dried biomass (physico-chemical treatment by heat and alkali or acid), which serve as control as it was the only lipid determination method in previous upstream studies (Schulze et al., 2014; Qian et al., 2019, 2020). The derivatization to FAME directly serves the purpose to produce biodiesel (Li et al., 2008; Sathish et al., 2014; Yousuf et al., 2017), whereas valuable FFA can be applied as nutritional supplements (Whigham et al., 2000; Smedman and Vessby, 2001; Kim et al., 2016).

The potential of new production hosts should be known as precisely as possible, therefore, it is necessary to evaluate product concentration and the effort and costs of processing. The aim is to classify different downstream processing methods in terms of their efficiencies and gentleness of lipid recovery using two new oleaginous yeasts.

## Materials and Methods

### Microorganisms

The oleaginous basidiomycetes examined in this study were newly screened and deposited at the DSMZ culture collection (Deutsche Sammlung von Mikroorganismen und Zellkulturen, Brunswick, Germany) as *Cryptococcus podzolicus* DSM 27192 and *Trichosporon porosum* DSM 27194 by Schulze et al. (2014). After genome sequencing and annotation both yeasts were phylogenetically reclassified to *S. podzolica* DSM 27192 (Aliyu et al., 2019) and *A. porosum* DSM 27194 (Gorte et al., 2019), respectively.

### Chemicals

All utilized chemicals were purchased either from Carl Roth GmbH & Co. KG (Karlsruhe, Germany) or Sigma-Aldrich Chemie GmbH (Taufkirchen, Germany) if not stated otherwise.

### Production of Single Cell Oil in Bioreactors

*Saitozyma podzolica* and *A. porosum* were cultivated in a 2.5 L Minifors bioreactor (Infors HT, Bottmingen, Switzerland) as described by Schulze et al. (2014). The cultivation of *S. podzolica* was performed at 22.5°C and pH 4, while *A. porosum* was grown at 25°C and pH 5. For SCO production, glucose was used as carbon source with initial concentration of 50 g/L. Each day, glucose was replenished manually to 90 g/L after determining the consumed carbon amount. After 96 h the cultivation broth was harvested in 50 mL aliquots by centrifugation at 4800 × *g* for 10 min. The supernatant was discarded and the biomass was preserved for 1 week at −20°C for downstream processing using BM, HPH, or U combined with chloroform-methanol extraction according to Bligh and Dyer (BD) and Folch (F), or by using ethanol-hexane (EH) as extractants.

### Nile Red Staining and Imaging

Nile red staining was applied according to the staining protocol of Silve et al. (2018). Yeast cells concentration was adjusted to 0.1 g/L in 1 mL of 50 mM potassium phosphate buffer pH 7.4 and centrifuged for 5 min at 6000 × *g*. Afterward, 200 μL of the supernatant was withdrawn and replenished with 200 μL of nile red stock solution (30 μg/mL in DMSO). The samples were mixed prior to a 10 min incubation period at 40°C. After that, the samples were washed with distilled water. For microscopic imaging the microscope Axioplan 2 (Zeiss, Jena, Germany) was used equipped with a × 63 LD Plan-Neofluar magnifying objective (Zeiss, Jena, Germany) and Axiocam HRc (Zeiss, Jena, Germany). For fluorescence imaging the fluorescence filter set 09 from Zeiss was used, i.e., the following filters: excitation BP 450–490, beam splitter FT 510, emission LP 515.

### Total Cell Count

The amount of total cells before and after disruption was visually counted under a microscope using Improved Neubauer (7178 05) counting chambers, consisting of large squares subdivided into 25 group squares of 0.04 mm^2^ area. The chamber’s depth is 0.1 mm. Six group squares were counted per each 1:1000 diluted sample. For total cell count calculation, the average amount of cells per group square (N) was determined and used for Eq. (1).

(1) $$T\,o\,t\,a\,l\,c\,e\,l\,l\,c\,o\,u\,n\,t\,\left[\frac{c\,e\,l\,l\,s}{m\,L}\right]=N\times D\,i\,l\,u\,t\,i\,o\,n\,\left(2.5\times 10^{5}\right)\,\frac{c\,e\,l\,l\,s}{m\,L}$$

### Cell Disruption Methods

For cell disruption the frozen biomass was thawed and washed twice with distilled water and resuspended in 50 mM potassium phosphate buffer pH 7.4 to a concentration of 100 g/L. After each disruption method the actual concentration of biomass was determined gravimetrically with a precision balance. In a pre-dried and pre-weighed 1.5 mL reaction tube 1 mL disrupted cells solution was provided and dried for 24 h at 100°C. Additionally, the same procedure was performed with 1 mL pure 50 mM potassium phosphate buffer. The weight of the buffer was subtracted from the weight of the biomass according to Eq. (2).

(2) W⁢e⁢i⁢g⁢h⁢t⁢(C⁢D⁢W)⁢[g]=D⁢i⁢s⁢r⁢u⁢p⁢t⁢i⁢o⁢n⁢s⁢u⁢s⁢p⁢e⁢n⁢s⁢i⁢o⁢n⁢[g]-B⁢u⁢f⁢f⁢e⁢r⁢[g]

#### High Pressure Homogenization (HPH)

The homogenizer EmulsiFlex-C3 (Avestin Europe GmbH, Mannheim, Germany) was used with a self-established continuous loop system by bridging the distance between the device outlet and the sample funnel with a 1 m silicone hose. During preliminary disruption experiments treatment time was optimized. HPH was processed in a volume of 15 mL of cell suspension at 2000 bar for 5 min (20 s per loop), resulting in 15 passes per sample. Subsequently, the sample was collected in a reaction tube on ice.

#### Bead-Milling (BM)

All BM experiments were performed using the bead mill MM 300 (Retsch GmbH, Haan, Germany) in 1.5 mL superspin microtubes (20170-030; VWR International GmbH, Darmstadt, Germany) with 425–600 μm acid washed glass beads (G8772; Sigma-Aldrich Chemie GmbH, Taufkirchen, Germany). Sample and glass beads were distributed in 1:1 ratio (v/v) in the microtubes. In preliminary experiments disruption time and frequency was optimized: the milling process was set at the frequency of 30 Hz/s for 20 min. Thereafter, the suspensions were pooled before extraction.

#### Ultrasonification (U)

Cell disruption by ultrasonification was operated with the 20 kHz ultrasonic homogenizer Sonopuls HD 3100 equipped with the probe MS 72 (Bandelin electronic GmbH & Co. KG, Berlin, Germany). The optimal amplitude, number of cycles and sonication time were determined in preliminary experiments. For the presented experiments, 30 ml cell suspension cooled on ice was sonicated using the maximum amplitude of 97% in a cycle of 50 s pulsing and 10 s pause for 3 × 10 min.

### Extraction Methods and Whole Lipid Determination

All extraction experiments were implemented in triplicates. 1 mL of disrupted cell suspension was used per extraction. As negative control, 1 mL of 50 mM potassium phosphate buffer pH 7.4 was processed.

### Chloroform Methanol Extractions

The miniaturized version of the methods of Folch (F) (Folch et al., 1957) and Bligh and Dyer (BD) (Bligh and Dyer, 1959), as adapted by Vasconcelos et al. (2018), were slightly modified in this study. F was performed by combining 1.9 mL potassium phosphate buffer, 1 mL disrupted cells suspension, 9.66 mL chloroform and 4.83 mL methanol. For BD 5.525 mL potassium phosphate buffer were added to 1 mL sample, followed by 7.25 mL chloroform and 7.25 mL methanol. The reaction tubes were inverted 20 times, covered in aluminum foil and were shaken for 30 min. Afterward, the phase separation was accelerated by centrifugation for 5 min and 1400 × *g*. To collect the entire lower chloroform phase, containing the lipids, a syringe and cannula was instrumentalised to puncture the interphase. The lipid phase was dispensed into preweighed glass tubes before complete evaporation of the chloroform in the vacuum concentrator Laborota 4000 (Heidolph Instruments GmbH & Co. KG, Schwabach, Germany) at 40°C, 700 × *g* and 10 mbar. For each method blank extractions containing buffer without biomass were performed to exclude artifactual results, as the used plastic consumables were slightly reactive to the solvents. To determine the whole cell lipid, the weight of the remaining lipids was detected gravimetrically and calculated according to Eqs (3) and (4).

(3) Lipid⁢extracted⁢[g]=L⁢i⁢p⁢i⁢d⁢c⁢r⁢u⁢d⁢e⁢[g]-B⁢l⁢a⁢n⁢k⁢[g]

(4) $$\%\,L\,i\,p\,i\,d\,s\,p\,e\,r\,W\,e\,i\,g\,h\,t\,\left(C\,D\,W\right)=\frac{L\,i\,p\,i\,d\,e\,x\,t\,r\,a\,c\,t\,e\,d\,\left[g\right]}{W\,e\,i\,g\,h\,t\,\left(C\,D\,W\right)\,\left[g\right]}\times 100\%$$

After weighting, the lipids were resuspended in 1 mL hexane and stored at −20°C prior to sample preparations for further analytical purposes.

#### Ethanol Hexane (EH) Solvent System

Ethanol hexane (EH) extraction of *S. podzolica* biomass was conducted as described in Silve et al. (2018) with slight modifications. 15.1 mL ethanol and 6.6 mL hexane were added to 1 mL disrupted cells suspension and mixed by inverting (20 times). The samples were wrapped in aluminum foil and shaken for 3 h at room temperature during which a monophasic suspension was formed. Cell debris was pelleted by centrifugation at 4800 × *g* for 10 min. Afterward, 10 mL were transferred to new reaction tubes and 5 mL ddH_2_O and 30 mL hexane were added to induce phase separation. After mixing for 3 min and centrifugation at 4800 × *g* for 10 min, 20 mL of the upper hexane phase was transferred to pre-weighed glass tubes before solvents were completely evaporated in the vacuum concentrator Laborota 4000 (Heidolph Instruments GmbH & Co. KG, Schwabach, Germany) at 40°C, 700 × *g* and 10 mbar. Similar to the other extraction methods, blank extractions without biomass were performed to exclude artifacts. The weight of the extracted lipids was determined with a precision balance and the whole cell lipid was calculated using Eqs (3) and (5).

(5) $$\%\mathrm{Lipids}\mathrm{per}\mathrm{Weight}\left(\mathrm{CDW}\right)=\frac{L\,i\,p\,i\,d\,e\,x\,t\,r\,a\,c\,t\,e\,d\,\left[g\right]}{W\,e\,i\,g\,h\,t\,\left(C\,D\,W\right)\,\left[g\right]}\times \frac{22.7\,{\left[m\,L\right]}^{*}}{10\,\left[m\,L\right]}\;\times \frac{32.9\,{\left[m\,L\right]}^{**}}{20\,\left[m\,L\right]}\times 100\%$$

^∗^Total volume monophase;

^∗∗^2.9 mL (ratio of hexane in 10 mL monophase) + 30 mL hexane (added for phase separation).

The extracted lipids were resolved in 1 mL hexane and stored at −20°C prior to sample preparations for further analytical purposes.

### Sample Preparation for Analytical Methods

#### Enzymatic Hydrolysis to Produce Free Fatty Acids (FFAs)

For enzymatic hydrolysis of the standard triglycerol trilinolein (T1388; TCI Deutschland GmbH, Eschborn, Germany) and extracted lipids, the method of Guauque Torres et al. (2014) was slightly adapted. 25 mg of trilinolein or extracted lipids were mixed with 0.65 g triton X-100, 1.25 mL TRIS-HCl buffer (pH 5.5), 0.5 mL distilled water and 90 mg of *Candida antarctica* lipase B (Novozym 435, Strem chemicals Europe, Kehl, Germany). The reaction mixture was incubated for 3 h in a NeoLab-rotator with vortex mixer (Heidelberg, Germany) at 50°C using program U2 at 20 rpm. Afterward 200 μL were used for HPLC analysis. All samples were processed in triplicates.

#### HPLC Analysis of Free Fatty Acids (FFAs)

Free fatty acids were determined by reversed-phase HPLC using a Kinetex EVO C18 (2.6 μm, 250 mm × 4.6 mm) from Phenomenex (Aschaffenburg, Germany). The HPLC system consisted of a pump, auto sampler and column oven from Agilent Technologies (Waldbronn, Germany). The fatty acids were separated in a binary gradient of acetonitrile (A) and water (B) with a flow rate of 1 mL/min. The injection volume was set to 10 μL and the temperature of the column oven to 50°C. The elution conditions were the following: 0–7 min isocratic 75A:25B, 7–17 min linear gradient up to 80A:20B, 17–22 min linear gradient up to 95A:5B, 22–30 min isocratic 95A:5B, followed by a reconditioning step of the column to 75A:25B for 5 min. FFAs were detected with an ELSD from Grace (Essen, Germany) at 38.1°C with a gas flow of 1.45 mL/min and the gain was set to 4.

#### Transesterification to Fatty Acid Methyl Esters (FAMEs)

For GC analysis, extracted SCOs were transesterified to FAMEs in a two phase system using two different catalysts. All experiments were performed in triplicates. Transesterification efficiency of both catalysts was determined by using 25 mg trilinolein as control.

##### Acidic transesterification

For acidic transesterification, 0.5 mL internal standard consisting of 2 mg/mL heptadecanoic acid and additional 0.5 mL hexane were added to 1 mL extracted lipids or 25 mg of standard in hexane. An equal volume of 2 mL 15% H_2_SO_4_ in methanol was added as catalyst. The reaction mixtures were incubated for 2 h at 100°C and 1000 rpm in a thermo-shaker (Universal Labortechnik, Leipzig, Germany). Samples were additionally mixed every 30 min by vortexing. To stop the reaction, the tubes were placed on ice for 10 min. To improve phase separation, 1 mL distilled water was added. The upper hexane phase containing FAMEs was transferred for GC analysis.

##### Alkaline transesterification

Alkaline transesterification of extracted lipids and 25 mg standard was done at 60°C and 1000 rpm for 20 min with 2 mL of 5% KOH in methanol as catalyst. 2 mg/mL of methyl benzoate was utilized as internal standard.

##### Direct transesterification

For direct transesterification (DT) the standard triglycerol trilinolein and biomass samples were processed. 15 ml of fresh biomass was taken at the end of the cultivation process and washed with 0.9% NaCl. The pellet was freeze-dried for 24 h at −30°C and 0.370 mbar using the BETA 1-8 freeze dryer (Christ, Osterode am Harz, Germany). 20 mg of freeze dried biomass or 25 mg of the trilinolein were applied. Subsequently, an acidic and alkaline transesterification was performed as described above.

#### GC Analysis of Fatty Acid Methyl Esters (FAMEs)

The quantitative and qualitative analysis of the transesterified FAMEs were performed gas-chromatographically using the 6890 N Network GC-System (Agilent Technologies Deutschland GmbH, Waldbronn, Germany). The device was coupled with a DB- Wax column (122–7032; l: 30 m d: 0.25 mm, Agilent Technologies Deutschland GmbH, Böblingen, Germany) and the detection was performed with a FID under 1.083 bar working pressure. 1 μL of sample was injected at the initial temperature of 40°C. The separation of the FAMEs was achieved by a temperature gradient from 40 to 250°C with a rate of 8°C/min and was kept for 10 min at 250°C. To identify and quantify the FAMEs the RM3 FAME Mix standard (07256-1AMP; Sigma Aldrich, Taufkirchen, Germany) was used.

### Statistical Analysis

Origin Software [version 2019 (9.6)] was used for statistical analysis. One-way ANOVA followed by *post hoc*-test Tukey were performed using *p*-value < 0.05.

## Results

Different combinations of mechanical cell disruption methods and solvent systems for lipid extraction were applied to both yeasts as outlined in Figure 1. Each step was evaluated for its efficiency and all combinations were subsequently compared with direct transesterification of freeze-dried biomass, which was used as the only analysis method for SCO production with these yeasts in former studies.

### Evaluation of Mechanical Cell Disruption Methods

**FIGURE 2:**
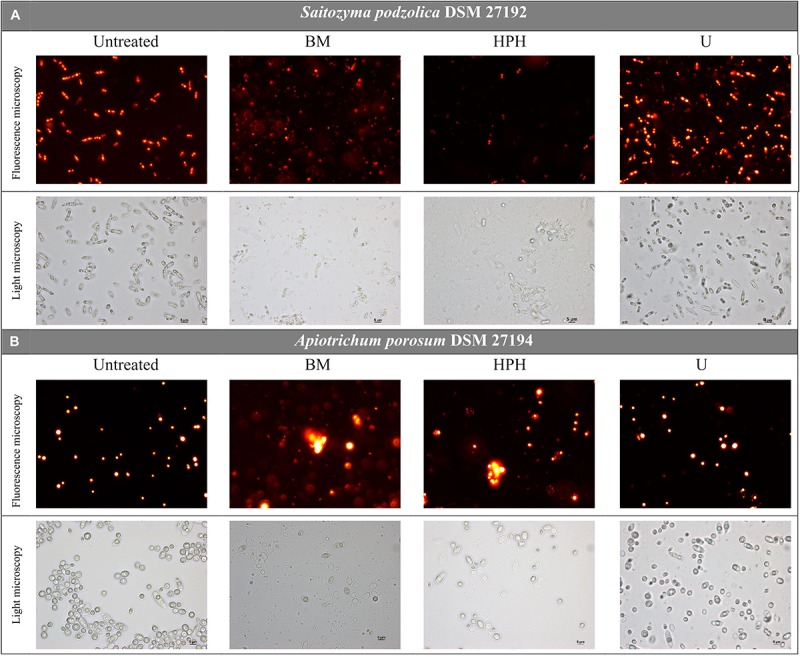
Light microscopy pictures of untreated and via BM, HPH and U mechanically disrupted, nile red stained yeast cells. **(A)** *S. podzolica*, **(B)** *A. porosum*. BM, bead mill; HPH, high pressure homogenization; U, ultrasonic treatment.

For both yeasts, disruption efficiencies of the three applied methods were optically determined and compared via nile red fluorescence assay and light microscopy prior to whole lipid extraction. Figure 2A provides microscopic images of untreated and disrupted cells of *S. podzolica*. After bead-milling (BM) and HPH mainly cell debris were observed, whereas after treatment with ultrasound (U) most of the cells seemed still to be intact. Total cell count confirmed the microscopical observation: after BM treatment 93% of the cells were found to be disrupted, 95% after HPH, but only 27% after treatment with U, although maximal amplitude was applied. Disruption of the cells probably enables lipid bodies to escape and to aggregate in aqueous buffer solution, which can be observed in the nile red images. Furthermore, HPH treatment might lead to emulsification by reduction of lipid droplet size as it is used for this purpose in the dairy industry. Therefore, fluorescence intensity of *S. podzolica* seems to be reduced after HPH treatment. The effect of the cell disruption approaches on *A. porosum* is shown in Figure 2B. Similar to *S. podzolica*, BM and HPH also seemed to be more efficient compared to U. However, all three treatments appeared to be less destructive to *A. porosum* than to *S. podzolica*, which was also confirmed by total cell count. After BM, HPH and U only 74, 53, and 8% of the cells were disrupted, respectively.

### Comparison of Whole Cell Lipid Extractions

After cell disruption whole lipid was extracted either by chloroform and methanol using two different protocols [according to Folch (F) and Bligh and Dyer (BD)] or by ethanol and hexane (EH). Latter extraction was only performed for *S. podzolica*. Whole cell lipid extraction yields per CDW are illustrated in Table 1. For *S. podzolica*, whole lipid yield and cell disruption efficiency are clearly correlated. Therefore, highest amounts of whole cell lipid were obtained after HPH, which was the most efficient cell disruption method, followed by BM and U regardless of the solvent system used for extraction. Consistently, extraction efficiency was always superior with EH, followed by BD and F for all cell disruption methods. The best method for whole cell lipid extraction of *S. podzolica* is therefore the combination of HPH and EH (46.9 ± 4.4%) which is 2.7 times higher than with the least favorable combination (U and F; 17.3 ± 3.1%). Observed differences are in most cases statistically significant, however, no significant difference was detected between HPH-EH and HPH-BD. Hence, both methods, HPH-BD and HPH-EH, are equally well-suited to achieve the best whole cell lipid yield for *S. podzolica*.

**TABLE 1:** Extracted whole cell lipid yields per cell dry weight (CDW) of *S. podzolica* and *A. porosum* after different cell disruption methods and solvents.

**Cell disruption method**	**Extraction method**	***S. podzolica***		***A. porosum***	
		**Disruption efficiency**	**Whole lipid per CDW [%]**	**Disruption efficiency**	**Whole lipid per CDW [%]**
BM	F	93%	27.9 ± 2.0^a^	74%	16.8 ± 2.7^a^
	BD		32.3 ± 2.5^b^		20.0 ± 3.2^a^
	EH		37.3 ± 3.4^c^		n.d.
HPH	F	95%	37.8 ± 2.3^c^	53%	15.0 ± 2.8^a^
	BD		43.4 ± 1.2^d^		14.1 ± 0.9^ab^
	EH		46.9 ± 4.4^d^		n.d.
U	F	27%	17.3 ± 3.1^e^	8%	n.d.
	BD		20.7 ± 3.0^e^		n.d.
	EH		n.d.		n.d.

*BM, bead mill; HPH, high pressure homogenization; U, ultrasonic treatment; F, extraction according to Folch; BD, extraction according to Bligh and Dyer; EH, ethanol-hexane extraction; n.d., not determined. All values are given as the mean ± standard deviation of three independent experiments. a, b, c, d, e indicates statistical differences (*p* = 0.05). ab indicates statistical difference between the disruption methods without changing the extraction method. Statistical analysis was performed separately for each yeast species.*

As expected from low disruption efficiencies, extracted whole cell lipid yields of *A. porosum* are also generally low (Table 1), i.e., lower than published lipid contents extracted by direct transesterification. The best combination was BM and BD with 20.0 ± 3.2%. Although highest whole lipid yields were also achieved from the more efficient cell disruption method (BM), trends are not as clear as for *S. podzolica* and observed differences are not statistically significant. The data for extracted whole cell lipid after U are not shown for *A. porosum* since cell disruption was not sufficient as already mentioned above.

### Determination of Optimal Fatty Acid Methyl Ester (FAME) Production

Quantification of lipids can be achieved indirectly via FAMEs by GC or in form of FFAs by HPLC. For both methods, sample preparation is necessary and might have an impact on (calculated) amount and fatty acid composition leading to underestimation of both lipid yield and quality.

**FIGURE 3:**
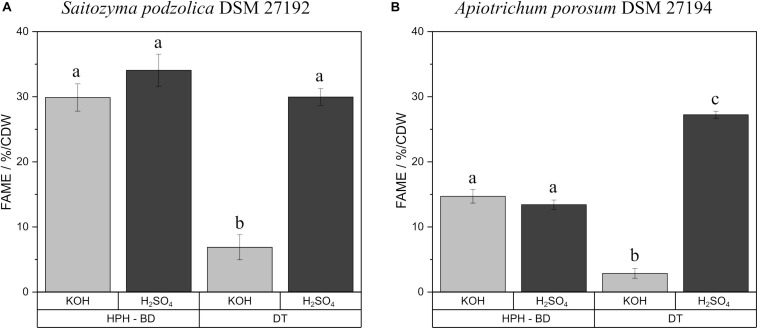
Comparison of KOH and H_2_SO_4_ catalysts for FAME production after HPH-BD treatment and direct transesterification of yeast biomass. % FAME per CDW from *S. podzolica* biomass is demonstrated in **(A)**, while **(B)** presents the same for *A. porosum*. The standard deviation of three independent experiments are indicated by the error bars. a, b, c illustrate statistical differences (*p* = 0.05). FAMEs, fatty acid methyl esters; BM, bead mill; HPH, high pressure homogenization; DT, direct transesterification; CDW, cell dry weight; BD, extraction according to Bligh and Dyer.

Transesterification of fatty acids derived from triacylglycerols/SCOs to FAMEs can be achieved by both acidic and alkaline methylation with H_2_SO_4_ or KOH as catalyst, respectively. For both methods transesterification efficiency and impact on polyunsaturated fatty acids were investigated by using the standard triacylglycerol trilinolein. FAME yield with KOH was 92.3 ± 2.8%, whereas, H_2_SO_4_ derivatization resulted in a FAME yield of 97.1 ± 3.1%. The results for transesterification of extracted lipids from biomass are shown in Supplementary Table S1. No significant difference was observed between F and BD extractions for both yeasts after transesterification. On that account, Figure 3 exemplifies the comparison with KOH and H_2_SO_4_ produced FAMEs after HPH-BD treatment and direct transesterification of yeast biomass. For *S. podzolica* (Figure 3A) both catalysts achieved an equal yield for prior disrupted and extracted lipids. For direct transesterified biomass the KOH technique is four times less efficient compared to acidic transesterification. Statistically, all three successful methods (disrupted and extracted lipids treated with KOH and H_2_SO_4_ and DT-H_2_SO_4_) are equally potent to achieve high FAME yields. Figure 3B likewise indicates no difference for *A. porosum* in transesterification catalyst for recovered lipids. Similarly to *S. podzolica*, alkaline direct transesterification is not convenient and nine times less potent compared to acidic technique for untreated biomass. Therefore, DT-H_2_SO_4_ is the most effective method to produce FAMEs from *A. porosum* biomass.

**FIGURE 4:**
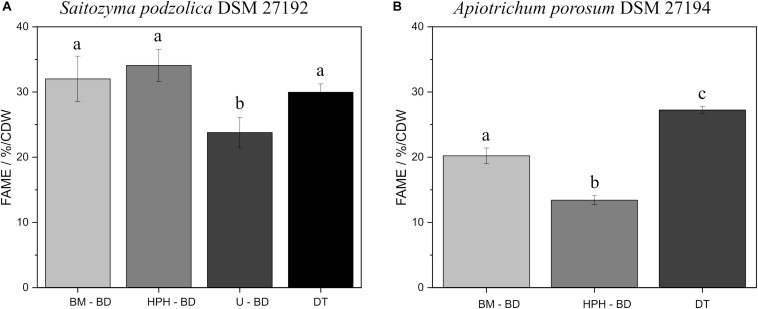
Comparison of all processed lipid recovery methods, using BD extraction and acidic transesterification, and acidic DT method. **(A)** % FAME per CDW from *S. podzolica* biomass. **(B)** % FAME per CDW from *A. porosum* biomass. The error bars result from the standard deviation of three independent experiments. a, b, c reveal statistical differences (*p* = 0.05). FAMEs, fatty acid methyl esters; CDW, cell dry weight; BM, bead mill; HPH, high pressure homogenization; DT, direct transesterification; BD, extraction according to Bligh and Dyer.

Since for processed lipids both catalysts are appropriate and for DT the acidic method is more adequate, Figure 4 contrasts the comparison of all disruption methods using BD extraction and DT after acidic transesterification. Figure 4A elucidates for *S. podzolica* with BM, HPH, and DT a statistically comparable FAME yield at approximately 30% per CDW. On the contrary, U is less efficient with 23.8 ± 2.3% FAME/CDW. However, EH after HPH is significantly the optimal method with the highest lipid yield (Supplementary Table S1). Different findings were observed for *A. porosum* (Figure 4B). By performing direct transesterification, the highest yield of 27.2 ± 0.5% FAME/CDW was detected. BM with 20.2 ± 1.2 and HPH with 13.4 ± 0.7% FAME/CDW are significant less sufficient methods to extract the lipids from this yeast.

### Enzymatic Hydrolysis of Extracted Lipids for Free Fatty Acid (FFA) Production

Enzymatic hydrolysis was used to convert triacylglycerols to FFA prior to HPLC analysis. Investigation of this method using trilinolein showed a conversion rate to FFA of 56.8 ± 2.6%. No statistical difference was observed between the extraction methods F and BD, whereas EH (37.0 ± 1.1% FFA/CDW with BM) was significantly more efficient than BD (18.0 ± 0.4% FFA/CDW with BM) and F (18.6 ± 0.5% FFA/CDW with BM). Cell disruption was shown to have a significant effect on FFA yields. BM (18.6 ± 0.5% FFA/CDW with F) and HPH (16.7 ± 0.9% FFA/CDW with F) resulted in significantly higher yields than U (11.2 ± 1.2% FFA/CDW with F) for *S. podzolica* (Supplementary Table S1).

For *A. porosum* BM (16.5% FFA/CDW) was a significantly more efficient method than HPH (7.7% FFA/CDW, Supplementary Table S1). The extraction methods had no statistical influence on the FFA yield. For both yeasts similar tendencies concerning the efficiencies of the investigated methods were observed for FFA with HPLC analysis as for FAME with GC.

### Impact of Cell Disruption, Extraction Methods, Transesterification, and Enzymatic Hydrolysis on Lipid Profiles

In order to judge which method is most suitable for extraction of oxidation sensitive polyunsaturated fatty acids, lipid profiles were determined. In terms of extraction systems, differences in lipid profiles were not observed when comparing the extraction methods F and BD (Supplementary Tables S2, S3). In contrast, extraction with EH resulted in significantly higher amounts of stearic acid and significantly lower amounts of linoleic acid.

With regard to cell disruption, no or only minor differences in lipid profile for *S. podzolica* biomass were detected when using HPH and BM (Supplementary Table S2). Analysis of the lipid profile of *A. porosum*, however, revealed significantly more linoleic acid and significantly less stearic acid for BM compared to HPH (Supplementary Table S3).

**FIGURE 5:**
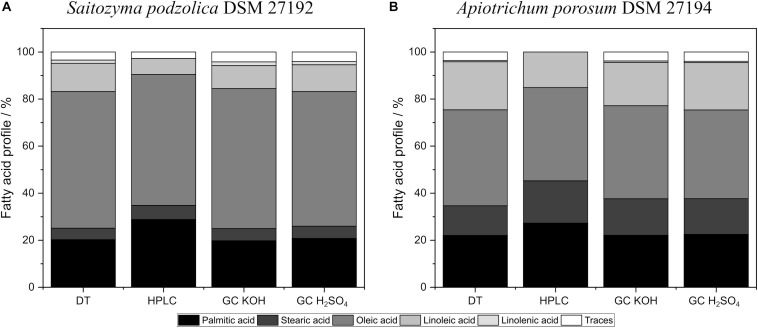
Fatty acid distribution of *in situ* transesterified and BM prior to BD processed biomass analyzed by HPLC and GC. **(A)** Fatty acid profiles in% per CDW of *S. podzolica*. **(B)** Fatty acid distribution in% per CDW of *A. porosum*. DT, direct transesterification; BD, extraction according to Bligh and Dyer.

Additionally, the investigated transesterification techniques did influence the lipid profiles for both yeasts. Figure 5 compares fatty acid profiles of both yeasts obtained by BM + BD and acidic direct transesterified biomass. Extracted and with KOH transesterified lipids of *S. podzolica* resulted in higher amounts of oleic acid and linolenic acid compared to lipids transesterified with H_2_SO_4_ (Figure 5A). However, lowest linoleic acid yields were detected with KOH transesterification of extracted lipids. No significant difference on lipid profile of DT and disrupted and H_2_SO_4_ treated lipids were observed for *S. podzolica*.

Similar to *S. podzolica*, *A. porosum* (Figure 5B) showed lower oleic acid yields for disrupted and H_2_SO_4_ handled lipids compared to DT or extracted and KOH treated lipids. For *A. porosum* linoleic acid yield is significant higher for DT than for the other disruption methods.

Comparison of FAME and FFA quantification indicates that yields for the saturated fatty acids, palmitic acid and stearic acid, are significantly higher and linoleic acid amounts are significantly lower represented in FFAs than in FAMEs for both yeasts. No significant difference was observed for oleic acid between FFAs and FAMEs with the exception that significantly higher oleic acid levels were detected with GC KOH for *S. podzolica*.

## Discussion

### Comparison of Cell Disruption Efficiencies

Mechanical cell disruption methods ensure cell wall disruption by forces of shear, abrasion and cavitation due to high pressure, velocity, heat, or sonication (Jacob, 1992; Ochsenreither et al., 2016; Probst et al., 2016). In this study the tendencies of all three cell disruption methods on wet, but frozen and thawed, biomass are similar for both yeasts, indicating HPH and BM as potent disruption methods, whereas U resulted to be least effective regardless of the fact, that the maximum amplitude was used. In literature sonication is known to be unsuitable for microorganisms inhabiting rigid and tough cell walls (Jacob, 1992; Yu et al., 2015; Probst et al., 2016). However, due to the different definitions and measurements a comparison of cell disruption methods in literature is difficult. For the ascomycete *Yarrowia lipolytica*, e.g., ultrasound turned out to be one of the most efficient techniques for lipid recovery (Zhang et al., 2014; Meullemiestre et al., 2016).

The cell wall structure of ascomycetous yeasts usually consists of two layers including an inner layer with polysaccharides and an outer layer bearing glycoproteins covalently bound to the inner layer. In contrast, for some basidiomycetes multilayered cell walls were observed (Van Der Klei et al., 2011). The difference in cell layer quantity may contribute to the higher cell wall rigidity of the basidiomycetes presented in this study compared to ascomycetous yeasts. HPH and BM enforce high shear stress to the cells, either by pressurizing cells through a small valve followed by striking a wall at high velocity in case of HPH or by impact with agitated grinding beads in case of bead-milling (Chisti and Moo-Young, 1986; Middelberg, 1995; Probst et al., 2016). The SCO of the oleaginous yeast *Cryptococcus curvatus* (Thiru et al., 2011) and the microalgae *Nannochloropsis* sp. (Halim et al., 2016) were recovered after HPH treatment, though yields of other disruption strategies were not compared. Whereas for *Y. lipolytica* (Meullemiestre et al., 2016) and for the yeast *C. curvatus*, the fungus *Mortierella isabellina* and the microalga *Chlorella sorokiniana* (Yu et al., 2015) among other methods BM was compared with U, resulting, similarly to this study, in higher effective cell disruption after BM.

Considering the total cell count and the microscopic images after disruption (Figure 2) *A. porosum* was less affected by mechanical disruption than *S. podzolica*. This might be due to differences in cell wall compositions of both yeasts. Since both yeasts are newly screened structural analysis of the cell walls does not exist in literature. In general, the yeast cell wall consists of heterogeneous and complex cross-linked polymers of oligosaccharides (glucan, mannoprotein, chitin). The highly elastic β-(1,3)- glucan chains and rigid β-(1,6)-glucan crosslinks contribute to the stability and firmness (Phaff, 1971; Jacob, 1992). In addition, a cell wall is reinforced in stationary growth phase by chitin crosslinking and increased mannoprotein binding (De Nobel et al., 1990; Touhami et al., 2003). Comparisons of cell wall composition and organization indicated high variabilities between different fungal organisms (Free, 2013). Especially remarkable is the ability of some species to form an exterior capsule of carbohydrate polymers (Doering, 2009), which are known to be produced by the members of the family *Trichosporonaceae* (Duarte-Oliveira et al., 2017) to which *A. porosum* belongs (Aliyu et al., 2020) and which might also contribute to the challenge to efficiently disrupt the cell.

The most effective lipid recovery method for *A. porosum* is the acid catalyzed direct transesterification of freeze-dried biomass, in which cell disruption, lipid extraction, and transesterification to FAMEs is combined in a single reaction under harsh conditions at 100°C for 2 h. In contrast, the direct transesterification with KOH using milder conditions (60°C for 20 min) turned out to be insufficient for direct transesterification, which proves the necessity of high temperature for direct FAME production for both tested yeasts. However, freeze drying as pre-treatment of the biomass is critical for this method. Moisture considerably decreases the yield of the direct transesterification (Sathish et al., 2014; Yousuf et al., 2017). The moisture content of wet oleaginous microorganisms is over 80% and therefore, higher than in oilseeds and needs to be removed (Ehimen et al., 2010; Jin et al., 2012). Consequently, freeze drying is absolutely necessary though it is the most energy consuming technique. For biodiesel, a low value product, this strategy contributes mainly to production costs.

In comparison, *S. podzolica* is easier to break with equal yields after derivatization of both wet biomass, disrupted using HPH and BM, and dry biomass using DT (Figure 4). In fact, this yeast is a potential candidate for biodiesel or oleochemical production. Cell disruption is the decisive step for SCO extraction in yeasts. At an efficiency of over 90% of disrupted cells according to total cell count (*S. podzolica*: 93 and 95% after BM and HPH, respectively) the conditions are suitable for subsequent extraction, at lower cell disruption efficiencies (*A. porosum*: 74% by BM and 53% after HPH), only a limited part of the SCO can be extracted.

### Energy Consumption of Biomass Pre-treatment and Cell Disruption

Depending on the purpose of the extracted lipid a suitable method for downstream processing can be chosen by taking the energy costs and labor into account. In Table 2 the amount of required energy for 1 kg of extracted SCO is presented for each performed downstream method and both yeasts. The energy consumption to extract whole lipid of *S. podzolica* is about 100 kWh/kg for all tested methods. This is derived from the extraction efficiency, disruption time and the method’s device power consumption, e.g., U is the least efficient but also the least power consuming method, therefore more biomass must be treated to reach 1 kg whole lipid resulting in comparable energy consumption in kWh/kg as with the other methods. However, the most energy efficient methods are BM and HPH prior the EH extraction with 96 and 95 kWh/kg, respectively. For whole lipid extraction from *A. porosum* nearly twice as much energy is required compared to *S. podzolica*, which is due to the low achieved cell disruption efficiency of this strain. On this account, *A. porosum* is less appropriate for potential industrial use.

**TABLE 2:** Comparison of energy consumption of extracted whole cell lipid and FAME production.

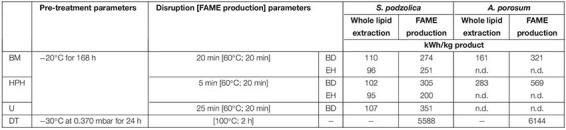

*Calculated data are extrapolations from the laboratory scale and energy consumption of laboratory devices. FAME, fatty acid methyl ester; BM, bead mill; HPH, high pressure homogenization; U, ultrasonic treatment; DT, direct transesterification; BD, Bligh and Dyer extraction; EH, ethanol-hexane extraction; n.d., not determined.*

Regarding the energy consumption for FAME production for application as biodiesel, the most energy saving method is HPH-EH-KOH for *S. podzolica* with 200 kWh/kg. For *A. porosum*, 60% more energy is needed for 1 kg FAME even when the most efficient method (BM-BD-KOH) for this yeast is used.

Comparing the energy consumption for FAME production with DT and mechanically treated biomass, DT is clearly the most energy consuming method. By performing HPH-EH-KOH with *S. podzolica* biomass just 3.6% of energy percentage is needed for one kg FAME compared to DT, for *A. porosum* biomass treated with BM-BD-KOH just 5.2% of DT’s energy amount is required. This fact results from the different required pre-treatment methods. Although the mechanically treated biomass was frozen for 168 h at −20°C, still less energy was consumed than for 24 h of vacuum freeze-drying. Consequently, freeze-drying is the most energy consuming technique, which is also confirmed by other studies (Meullemiestre et al., 2016). By considering the extrapolated data from laboratory scale in this study, *S. podzolica* is more suitable for potential industrial application for both, whole lipid recovery for oleochemicals as well as for transesterification for biodiesel production.

Within this study a higher SCO production potential of the yeast *S. podzolica* is proven, compared to the previous studies performing only DT with this yeasts’ biomass (Schulze et al., 2014; Qian et al., 2019, 2020). Moreover, the downstream process using HPH and ethanol-hexane extraction prior to transesterification is not only more efficient but also more energy saving than DT. However, there is still optimization potential regarding energy efficiency, e.g., shorter pre-treatment times and higher biomass scales need to be investigated.

### Influence of Solvents on Extraction Yields

Oleaginous yeasts produce neutral lipids, such as triacylglycerols as major component, di- and mono-acylglycerols, FFAs and sterols. These molecules are accumulated in lipid bodies surrounded by polar phospholipids and serve for energy storage. Polar lipids, like phospholipids, sphingolipids and glycolipids, are incorporated in the flexible cell membrane (Brown, 2001; Breil et al., 2017). To recover the whole lipid of microorganisms generally a system of polar and non-polar solvents is applied. The polar cosolvent is needed to break up the protein layer surrounding the lipid droplets in order to make the lipids accessible to the non-polar solvent. The most famous and efficient standard techniques are according to Folch (Folch et al., 1957) and Bligh and Dyer (Bligh and Dyer, 1959) using the solvents chloroform and methanol. However, chloroform is a highly toxic and carcinogenic solvent and its usage is therefore prohibited in the food industry (Grima et al., 1994; Breil et al., 2016). In the food industry, EH is used as low-toxicity alternative in lipid extraction of oil seeds and canola (Grima et al., 1994; Biondo et al., 2015). This extraction method is also well-established for algal lipids (Silve et al., 2018). For yeast cells, however chloroform-methanol extraction is still the most common method even though Èertík et al. (1996) already showed that EH is also suitable for *Mucor mucedo*. In contrast to chloroform, hexane is non-carcinogenic but still neurotoxic (Joshi and Adhikari, 2019). After all, there is no alternative to hexane in lipid extraction (Breil et al., 2016). For this reason, the less harmful solvents ethanol and hexane were also considered for lipid extraction in the present study.

Analyzing the extraction techniques on whole lipid extraction, BD was more effective compared to F for *S. podzolica* probably because the ratio of the polar methanol is higher in BD leading to a more efficient extraction of polar lipids (Dong et al., 2016; Vasconcelos et al., 2018). For *A. porosum* both extraction methods showed no significant differences, presumably due to the insufficient cell disruption. After transesterification, however, F und BD extraction revealed the same yield of transesterifiable lipids for both yeasts proving an equal ability of the methods to recover neutral lipids.

The less harmful EH solvent mixture turned out to be highly suitable for SCO purification of *S. podzolica*. After HPH treatment EH extraction proved to be similarly potent as BD extraction and the highest whole cell lipid yields were achieved. Remarkably, after derivatization of the HPH treated biomass the highest FAME yields resulted from EH extracted lipids with 46.0 ± 6.8% FAME/CDW. Compared to the former standard analysis DT (30.0 ± 1.3% FAME/CDW) with this new approach 53% increase of production potential of *S. podzolica* was reached. Since HPH is the most effective disruption method for *S. podzolica*, here the highest amount of lipids was accessible for extraction. Hexane has a lower polarity than chloroform, consequently neutral lipids have a higher affinity to non-polar solvents (Cooney et al., 2009). Thus, more transesterifiable acylglycerols and FFAs should be accessible for EH extraction. Additionally, the EH method was completed in 3 h, whereas BD was carried out in 30 min. The longer extraction time might also contribute to this result. In literature ethanol-hexane extractions are commonly used for microalgae lipid recovery with likewise high extraction effectiveness (Grima et al., 1994; Silve et al., 2018). For the yeast *Lipomyces kononenkoae*, also the less polar solvent toluene revealed higher extraction yields over chloroform (Vasconcelos et al., 2018). Hence, hazardous chloroform methanol mixtures can be dispensed with less harmful ethanol hexane system without scarifying oil recovery for *S. podzolica*.

### Enzymatic Hydrolysis of Triglycerides

The linoleic acid yield of trilinolein hydrolysis was 56.8% in this study. *Candida antarctica* lipase B is reported to cleave triglycerides selectively in position sn-1 and sn-3 (Rogalska et al., 1993; Villeneuve et al., 1995; Watanabe et al., 2015). For long chain fatty acids *Candida antarctica* lipase B has a low sn-1 selectivity and for short chain fatty acids a low sn-3 selectivity compared to a high typoselectivity for short chain fatty acids (Villeneuve et al., 1995). Therefore, enzymatic cleavage of a triglyceride leads to one monoglyceride and two FFAs under the applied conditions. Consequently, 85% of the theoretical yield are reached in this study. Theoretical and experimental FAME yield is higher than FFA yield, respectively. Therefore, FFA production is only economically feasible for high value products that are incompatible with FAME, e.g., in nutraceuticals as linoleic acid showed antiobesity, antiatherosclerosis, anticancerogenic, and immunomodulating effects *in vitro* and in animal studies (Whigham et al., 2000; Smedman and Vessby, 2001; Kim et al., 2016). The side products, mono acylglycerides, of the applied enzymatic fatty acid production with *Candida antarctica* lipase B from SCO are of interest for the food industry as they are widely applied as emulsifiers, e.g., in bakery products (Mettler and Seibel, 1993).

### Impact of Enzymatic Hydrolysis, Transesterification, and Cell Disruption on Lipid Profiles

Comparison of FAME lipid profiles and FFA lipid profiles revealed a higher content of saturated fatty acids for FFA than for FAME. This might be due to the enzymatic hydrolysis with *Candida antarctica* lipase B, as this enzyme is reported to hydrolyze stearic acid faster than oleic acid, linoleic acid or linolenic acid (Nalder et al., 2014). In the case of unsaturated acids, linolenic acid is preferred to linoleic acid and oleic acid (Nalder et al., 2014). This could not be confirmed in our study as the quantification of linolenic acid was not possible due to co-elution with the emulsifier used in the reaction.

Alkaline transesterification methods are recommended for lipid analysis as they are faster, more efficient and the reaction is in general more complete than acidic transesterification (Christie, 1993; Liu, 1994; Aldai et al., 2005; Carlini et al., 2014). Another limitation of sulfuric acid catalyzed transesterification, especially for up-scaling, is possible corrosion of reaction vessels due to salt interactions (Carlini et al., 2014). However, with the alkaline methods only transesterification is possible but not the esterification of FFAs. Instead, the acidic catalysis is suitable for both esterification and transesterification (Liu, 1994; Aldai et al., 2005). Another difference between alkaline and acidic transesterification is that *N*-acyl lipids are not transesterified by alkaline methods but only by acid catalysis (Mayberry, 1981; Galbraith and Wilkinson, 1991; Christie, 1993). A disadvantage of acidic transesterification is that this method could cause isomerization and methoxy artifacts in conjugated fatty acids (Aldai et al., 2005). However, Yamasaki et al. (1999) showed sulfuric acid catalyzed transesterification is a relatively mild method as 71% of conjugated linoleic acid are not isomerized after 120 h of methylation (Yamasaki et al., 1999). Therefore, differences in lipid profiles between alkaline catalysis and acid catalysis might be more likely due to esterification of FFAs and *N*-acyl lipids with the acidic method, whereas these are not quantified with alkaline transesterification. However, the total amount of FAME is the same for both methods, as alkaline triglyceride transesterification might be more complete and therefore compensate the non-methylated FFAs and *N*-acyl lipids in regards to total FAME amount.

Significant differences in lipid profile of *A. porosum* treated with BM and HPH were observed. After HPH linoleic acid content was significantly lower than after BM. To our knowledge, there are no studies existing addressing the lipid profiles of yeasts in relation to cell disruption methods. The influence of BM on fatty acid composition was addressed only in a single publication where no differences in lipid composition of *Chlorella vulgaris* after BM compared to grinding, ultrasonication, enzymatic lysis, and microwaves was observed (Chi et al., 2011). Studies on the influence of HPH on the fatty acid composition of various foods provide different results. Thus, Rodríguez-Alcalá et al. (2009) reported no influence of HPH on fatty acid composition of milk up to a pressure of 3500 bar. In tomato juice an increase in the oxidation product of unsaturated fatty acids, *n*-hexanal, was reported after HPH treatment at a pressure of 5000 bar (Porretta et al., 1995). While Kuhn and Cunha (2012) observed a significant increase in primary oxidation products due to HPH treatment of whey protein isolate stabilized oil in water emulsions of flaxseed oil already at a pressure of 800 bar. Therefore, the differences in linoleic acid content after BM and HPH treatment might be due to oxidation of linoleic acid during HPH treatment.

## Conclusion

The aim of the presented work was to investigate the impact of cell disruption, extraction, and transesterification methods as well as enzymatic hydrolysis on fatty acid extraction yields and their lipid profile of SCO of two unconventional yeasts, *S. podzolica* and *A. porosum*, to assess their potential economic profitability. It was shown that BM and HPH were the most appropriate cell disruption methods for *S. podzolica* whereas DT was best for *A. porosum* followed by BM. No differences could be observed between F and BD as extraction methods for derivatized lipids, while the EH system was superior for *S. podzolica*, which is less harmful in comparison to the highly toxic chloroform-methanol mixture. With regard to the lipid profiles, differences between BM and HPH were observed, which might be due to oxidation of linoleic acid while HPH processing. Comparison of alkaline and acidic transesterification of recovered oil revealed higher linoleic acid yield for H_2_SO_4_ catalysis. Both are similarly potent transesterification methods for recovered oil, whereas direct transesterification needs to undergo harsh conditions provided by this study’s acidic transesterification.

We are the first to evaluate the downstream process properties of this new SCO producing yeasts for potential industrial use like biodiesel or oleochemical production. Concluding from this, *A. porosum* is less suitable compared to *S. podzolica*. The oil of *A. porosum* is more difficult to recover and more efficient with high energy consuming pre-treatment process. However, for *S. podzolica* a 53% higher production potential was shown using HPH-EH prior to transesterification, compared to the former standard analysis DT.

## Data Availability Statement

All data generated or analyzed during this study are included in this published article and its additional file.

## Author Contributions

OG and RH designed and planned the study, analyzed the data and drafted the article supervised by KO. OG performed yeasts cultivation, oil recovery, and GC analysis. RH conducted the enzymatic hydrolysis and the statistical analysis. OG and IP performed the nile red assay. CSt enabled the use of the bead mill. AS, WF, and CSy constructively contributed to the content. All authors critically revised the article.

## Conflict of Interest

The authors declare that the research was conducted in the absence of any commercial or financial relationships that could be construed as a potential conflict of interest.

## Abbreviations

BD: Bligh and Dyer
BM: bead milling
CDW: cell dry weight
EH: ethanol hexane extraction system
ELSD: evaporative light scattering detector
F: Folch
FAME: fatty acid methyl ester
FFA: free fatty acid
FID: flame ionization detector
GC: gas chromatography
HPLC: high performance liquid chromatography
HPH: high pressure homogenization
SCO: single cell oil
U: ultrasonification.
