# Supplementary material for: Evaluation of Downstream Processing, Extraction, and Quantification Strategies for Single Cell Oil Produced by the Oleaginous Yeasts Saitozyma podzolica DSM 27192 and Apiotrichum porosum DSM 27194
Source: Front Bioeng Biotechnol. 2020 Apr 24;8:355. doi: 10.3389/fbioe.2020.00355 (PMC7193083; doi:10.3389/fbioe.2020.00355)
Supplement: Supplementary file 1 [file Table_1.DOCX]

Table S1: Recovered lipid yields of methylated and free fatty acids from S.podzolica and A. porosum biomass.

|  |  | S. podzolica | | | A. porosum | | |
| --- | --- | --- | --- | --- | --- | --- | --- |
|  |  | % FAME/ CDW | | % FFA/ CDW | % FAME/ CDW | | % FFA/ CDW |
|  |  | KOH | H_2_SO_4_ | *C. antarica* lipase B | KOH | H_2_SO_4_ | *C. antarica* lipase B |
| BM | F | 30.7 ± 0.6 ^a^ | 30.1 ± 1.5 ^a^ | 18.6 ± 0.5 ^a^ | 23.4 ± 0.5 ^a^ | 19.6 ± 1.0 ^b^ | 16.5 ± 1.5 ^a^ |
|  | BD | 30.1 ± 1.5 ^a^ | 32.0 ± 3.5 ^a^ | 18.0 ± 0.4 ^a^ | 23.8 ± 1.1 ^a^ | 20.2 ± 1.2 ^b^ | 16.5 ± 0.5 ^a^ |
|  | EH | 32.9 ± 1.7 ^a^ | 28.9 ± 1.0 ^a^ | 37.0 ± 1.1 ^b^ | n.d. | n.d. | n.d. |
| HPH | F | 30.4 ± 0.9 ^a^ | 29.1 ± 1.3 ^a^ | 16.7 ± 0.9 ^a^ | 13.9 ± 1.5 ^c^ | 14.8 ± 1.2 ^c^ | 7.7 ± 0.1 ^b^ |
|  | BD | 29.9 ± 2.1 ^a^ | 34.1 ± 0.1 ^a^ | 16.9 ± 2.0 ^a^ | 14.7 ± 1.1 ^c^ | 13.4 ± 0.7 ^c^ | 7.6 ± 0.3 ^b^ |
|  | EH | 46.0 ± 6.8 ^b^ | 44.2 ± 4.0 ^b^ | 32.4 ± 1.6 ^c^ | n.d. | n.d. | n.d. |
| U | F | 19.0 ± 0.4 ^c^ | 25.7 ± 1.8 ^d^ | 11.2 ± 1.2 ^d^ | n.d. | n.d. | n.d. |
|  | BD | 19.8 ± 0.8 ^c^ | 23.8 ± 2.3 ^c^ | 12.0 ± 0.2 ^d^ | n.d. | n.d. | n.d. |
| DT | - | 6.9 ± 1.9 ^e^ | 30.0 ± 1.3 ^a^ | n.d. | 2.9 ± 0.8 ^d^ | 27.2 ± 0.5 ^e^ | n.d. |

The standard deviation of three independent experiments is indicated. FAME: fatty acid methyl ester; FFA: free fatty acid; CDW: cell dry weight; BM: bead mill; HPH: high pressure homogenization; U: ultrasonic treatment; DT: direct transesterification; F: extraction according to Folch; BD: extraction according to Bligh and Dyer; EH: ethanol-hexane extraction. a, b, c, d, e indicate statistical differences (p=0.05). ab indicates statistical difference between the disruption methods without changing the extraction method. Statistical analysis was performed separately for each yeast species and separately for FAME and FFA.

Table S2: Lipid profiles of *S. podzolica* DSM 27192 biomass after derivatisation of each downstream processing method. The indicated standard deviation comprises of three independent analyses

|  | GC  % FAME/CDW | | | | | | | | | | | | | | | | HPLC  % FFA/ CDW | | | | | | | | DT  % FAME/ CDW | |
| --- | --- | --- | --- | --- | --- | --- | --- | --- | --- | --- | --- | --- | --- | --- | --- | --- | --- | --- | --- | --- | --- | --- | --- | --- | --- | --- |
|  | BM | | | | | | HPH | | | | | | U | | | | BM | | | HPH | | | U | |  |  |
|  | F | | BD | | EH | | F | | BD | | EH | | F | | BD | | F | BD | EH | F | BD | EH | F | BD |  |  |
|  | KOH | H_2_SO_4_ | KOH | H_2_SO_4_ | KOH | H_2_SO_4_ | KOH | H_2_SO_4_ | KOH | H_2_SO_4_ | KOH | H_2_SO_4_ | KOH | H_2_SO_4_ | KOH | H_2_SO_4_ |  |  |  |  |  |  |  |  | KOH | H_2_SO_4_ |
| Palmitic acid | 19.8 ±0.0 | 20.8 ±0.0 | 19.8 ±0.1 | 20.8 ±0.0 | 20.4 ±0.1 | 21.5 ±0.1 | 20.0 ±0.1 | 20.5 ±0.1 | 19.9 ±0.0 | 20.6 ±0.0 | 20.5 ±0.1 | 21.3 ±0.1 | 20.0 ±0.1 | 20.5 ±0.1 | 19.9 ±0.1 | 20.6 ±0.1 | 28.2  ±1.5 | 28.8  ±0.2 | 21.1  ±0.3 | 28.7  ±1.3 | 27.7  ±1.0 | 19.2  ±1.0 | 22.6  ±0.5 | 23.4  ±0.5 | 19.7 ±0.1 | 20.3 ±0.2 |
| Stearic acid | 5.3 ±0.1 | 5.2 ±0.1 | 5.2 ±0.1 | 5.2 ±0.2 | 5.4 ±0.0 | 5.7 ±0.0 | 5.2 ±0.0 | 4.8 ±0.0 | 5.16 ±0.0 | 5.0 ±0.1 | 5.6 ±0.1 | 5.4 ±0.0 | 5.0 ±0.0 | 3.4 ±2.2 | 5.0 ±0.0 | 4.7 ±0.1 | 6.6  ±0.3 | 6.0  ±0.2 | 7.4  ±0.8 | 5.1  ±1.0 | 5.1  ±0.5 | 7.3  ±0.3 | 6.3  ±0.4 | 5.6  ±0.1 | 4.7 ±0.2 | 4.9 ±0.0 |
| Oleic acid | 59.4 ±0.0 | 57.0 ±0.4 | 59.5 ±0.2 | 57.2 ±0.2 | 60.6 ±0.1 | 57.5 ±0.2 | 59.3 ±0.1 | 57.0 ±0.3 | 59.5 ±0.0 | 57.4 ±0.2 | 60.8 ±0.2 | 58.6 ±0.3 | 59.2 ±0.3 | 58.1 ±2.4 | 59.0 ±0.1 | 56.5 ±0.3 | 56.9  ±1.0 | 55.7  ±2.2 | 58.3  ±0.5 | 54.9  ±1.9 | 56.9  ±1.6 | 58.7  ±1.3 | 56.8  ±1.3 | 57.9  ±3.7 | 63.2 ±0.6 | 58.0 ±0.1 |
| Linoleic acid | 9.8 ±0.0 | 11.9 ±0.2 | 9.8 ±0.0 | 11.4 ±0.2 | 8.2 ±0.2 | 9.4 ±0.2 | 9.9 ±0.0 | 12.0 ±0.2 | 9.9 ±0.0 | 11.5 ±0.2 | 7.6 ±0.2 | 8.7 ±0.1 | 10.8 ±0.1 | 12.5 ±0.2 | 10.6 ±0.0 | 12.6 ±0.2 | 7.6  ±0.3 | 6.8  ±0.2 | 6.0  ±0.1 | 6.9  ±0.3 | 6.3  ±0.3 | 5.5  ±0.1 | 7.1  ±1.0 | 7.4  ±1.3 | 10.6 ±0.1 | 12.1 ±0.1 |
| Linolenic acid | 1.6 ±0.0 | 1.3 ±0.0 | 1.6 ±0.0 | 1.4 ±0.0 | 1.6 ±0.0 | 1.5 ±0.0 | 1.6 ±0.0 | 1.4 ±0.0 | 1.6 ±0.0 | 1.4 ±0.0 | 1.7 ±0.0 | 1.5 ±0.0 | 1.6 ±0.0 | 1.3 ±0.0 | 1.5 ±0.0 | 1.3 ±0.0 | n.d. | n.d. | n.d. | n.d. | n.d. | n.d. | n.d. | n.d. | 1.3 ±0.0 | 1.4 ±0.0 |
| Traces | 4.1 | 3.8 | 4.2 | 4.1 | 3.8 | 4.5 | 4.0 | 4.4 | 4.0 | 4.2 | 3.7 | 4.3 | 3.6 | 4.2 | 4.0 | 4.4 | 1.5  ±2.5 | 2.7  ±2.4 | 7.2  ±0.2 | 4.4  ±0.2 | 4.0  ±0.5 | 9.3  ±2.2 | 7.3  ±0.8 | 5.7  ±0.2 | 0.5 | 3.4 |

FAME: fatty acid methyl ester; FFA: free fatty acid; CDW: cell dry weight; BM: bead mill; HPH: high pressure homogenization; U: ultrasonic treatment; DT: direct transesterification; F: extraction according to Folch; BD: extraction according to Bligh and Dyer; EH: ethanol-hexane extraction; n. d.: not determined.

Table S3: Lipid profiles of recovered and transesterified or hydrolyzed SCO of *A. porosum* DSM 27194. The indicated standard deviation comprises of three independent analyses.

|  | GC  % FAME/ CDW | | | | | | | | HPLC  % FFA/ CDW | | | | DT  % FAME/ CDW | |
| --- | --- | --- | --- | --- | --- | --- | --- | --- | --- | --- | --- | --- | --- | --- |
|  | BM | | | | HPH | | | | BM | | HPH | |  |  |
|  | F | | BD | | F | | BD | | F | BD | F | B |  |  |
|  | KOH | H_2_SO_4_ | KOH | H_2_SO_4_ | KOH | H_2_SO_4_ | KOH | H_2_SO_4_ |  |  |  |  | KOH | H_2_SO_4_ |
| Palmitic acid | 22.4 ±0.5 | 22.5 ±0.1 | 22.1 ±0.0 | 22.5 ±0.1 | 22.1 ±0.2 | 23.2 ±0.1 | 22.0 ±0.1 | 23.2 ±0.1 | 27.4  ±0.4 | 27.3  ±0.2 | 26.3  ±0.0 | 26.1  ±0.3 | 22.7 ±0.6 | 22.0 ±0.5 |
| Stearic acid | 15.7 ±0.3 | 15.2 ±0.0 | 15.5 ±0.1 | 15.2 ±0.1 | 17.6 ±0.0 | 15.9 ±0.1 | 17.5 ±0.1 | 15.9 ±0.1 | 18.3  ±0.4 | 18.0  ±0.2 | 19.3  ±0.5 | 18.6  ±0.2 | 14.4 ±2.3 | 12.6 ±1.6 |
| Oleic acid | 40.01±0.7 | 37.6 ±0.2 | 39.6 ±0.1 | 37.7 ±0.2 | 39.0 ±0.2 | 37.2 ±0.2 | 39.2 ±0.0 | 37.2 ±0.2 | 39.9  ±0.5 | 39.6  ±0.1 | 40.8  ±0.6 | 41.8  ±0.2 | 43.3 ±1.3 | 40.8 ±1.7 |
| Linoleic acid | 18.8 ±0.3 | 20.3 ±0.1 | 18.5 ±0.1 | 20.1 ±0.2 | 17.0 ±0.1 | 19.3 ±0.1 | 17.0 ±0.1 | 19.4 ±0.2 | 14.4  ±0.5 | 15.1  ±0.3 | 13.6  ±0.2 | 13.5  ±0.3 | 19.7 ±0.5 | 20.4 ±0.3 |
| Linolenic acid | 0.6 ±0.01 | 0.6 ±0.0 | 0.6 ±0.0 | 0.6 ±0.0 | 0.7 ±0.0 | 0.6 ±0.0 | 0.6 ±0.0 | 0.6 ±0.0 | n.d. | n.d. | n.d. | n.d. | n.d. | 0.5 ±0.1 |
| Traces | 3.5 | 3.9 | 3.8 | 3.9 | 3.7 | 3.8 | 3.7 | 3.8 | n.d. | n.d. | n.d. | n.d. | n.d. | 3.6 |

FAME: fatty acid methyl ester; FFA: free fatty acid; CDW: cell dry weight; BM: bead mill; HPH: high pressure homogenization; U: ultrasonic treatment; DT: direct transesterification; F: extraction according to Folch; BD: extraction according to Bligh and Dyer; EH: ethanol-hexane extraction; n. d.: not determined.
